# From Land to Water: Taking Fish Welfare Seriously

**DOI:** 10.3390/ani10091585

**Published:** 2020-09-05

**Authors:** Walter Sánchez-Suárez, Becca Franks, Lauri Torgerson-White

**Affiliations:** 1 Department of Research, Mercy for Animals, Los Angeles, CA 90046, USA; lauritorgerson@gmail.com; 2 Department of Environmental Studies, New York University, New York City, NY 10003, USA; beccafranks@gmail.com

**Keywords:** fish welfare, positive welfare, aquaculture, animal welfare

## Abstract

**Simple Summary:**

Our knowledge of fish welfare is still scant relative to the enormous demands created by aquaculture expansion and focuses primarily on preventing poor health without considering provision of environments conducive to positive experiences. We are far from understanding what individuals of the different species classified under the umbrella term “fish” need to experience good lives. The aquaculture industry has been expanding swiftly, and fishes belonging to hundreds of very different species are now farmed by the billions. Informed by terrestrial animal welfare science, this article aims to set a framework for making progress in investigating how to provide farmed fish with good welfare.

**Abstract:**

This article aims to use contemporary (terrestrial) animal welfare science as a lens to evaluate the state of knowledge concerning welfare in fish species, focusing on farmed fishes. We take advantage of the vast expertise—including previous pitfalls and accomplishments—in the investigation of welfare in terrestrial vertebrates, borrowing questions and methodologies from terrestrial animal welfare science in order to (1) better understand the challenges and opportunities in the study of welfare in fish species, and (2) propose strategies for filling knowledge gaps.

## 1. Introduction

The global aquaculture industry has been expanding at an unprecedented rate. Figures reveal that in 2015 the number of individual farmed fishes probably exceeded the number of chickens produced for meat, the most farmed terrestrial vertebrates [1,2,3]. An estimated 51 to 167 billion farmed fishes were slaughtered for food in 2017, a 4–6% increase from 2015 [4]. Unlike chickens farmed for meat (a single species), farmed fishes span hundreds of species that live in different environments and have different needs. Thus, we are far from understanding their unique and specific welfare requirements. As it was the case with chickens and other farmed terrestrial animals, we now have key evidence supporting the hypothesis that fishes are sentient and, therefore, deserving of moral consideration. Filling the gaps in our knowledge of their welfare is urgently needed to ensure that harms against them are minimized and our ethical responsibilities are met. 

## 2. An Updated Concept of Animal Welfare

Evaluating the current state of welfare science for farmed fish species requires a clear, contemporary discussion framework. Donald Broom provided an early and insightful definition of animal welfare in 1986: “The welfare of an animal is its state as regards its attempts to cope with its environment” [5], that is, how successful is an animal when trying to cope with her or his environment”. Later, Marian Dawkins developed complementary operational criteria: “Improvements in animal welfare can be based on the answers to two questions: Q1: Will it improve animal health? and Q2: Will it give the animals something they want?” [6]. Most recently, animal welfare scientists expanded on these foundations by recognizing the need to aim beyond a mere reduction of suffering and have instead begun to explore what might constitute a “good life” for individuals of a particular species [7,8]. Interest in this good-life/positive-welfare approach has surged in recent years, with numerous reviews on the topic published in the last year alone [9,10,11,12,13,14,15,16,17,18,19].

Prior to this most recent development, much of animal welfare had focused on alleviating negative states, such as fear, pain, and frustration. While the precise contours of the good-life/positive-welfare approach are still being developed, at its core, this new wave of scholarship acknowledges that negative framing will not lead to satisfactory welfare outcomes and that the range of topics explored by animal welfare scientists must be expanded [10,19]. Interestingly, a similarly radical frameshift is evident in human psychology [20]. The “positive psychology” literature includes the important insight that, in addition to understanding psychological distress and pathologies, we must ask questions about what makes a life “worth living” [8]. Moreover, recent work in positive psychology stresses that a good life involves more than simply feeling “good” emotions all the time; a more dynamic and complex view is required [21]. Lomas and Ivtzan, in their paper on human psychology, write that “there is a movement away from a simplistic binary view that unreservedly classifies phenomena as either positive and negative, valorizing the former while condemning the latter, towards a more nuanced appreciation of the dialectical complexities of flourishing” [12]. Similar patterns are evident in nonhuman animal welfare as well. For example, in addition to wanting good material outcomes (e.g., good food, adequate shelter), many animals want to engage in challenges [22] and decision-making (e.g., learning, choosing) [23,24]. According to years of literature on animal welfare in zoos [25], agency, choice, control, cognitive stimulation, meaning, and challenge are crucial welfare dimensions to consider [19,26,27,28,29].

In sum, this brief history of terrestrial animal welfare science cautions that biological health and alleviation of negative states are in most cases insufficient to prevent poor welfare. Thus, welfare schemes must focus not only on coping and good health but on in-depth knowledge of a species’ full behavioral repertoire and what individual members of the species may want. Within this framework, animal welfare must be understood as “a continuum that varies from very good to very poor” [30]. In turn, this framework emphasizes that being healthy does not imply good welfare—that is, good health is a necessary but not sufficient prerequisite in the good-life equation, a consideration that is crucial to advancing fish welfare. These observations set the basic criteria for evaluating the current state of fish welfare in aquaculture.

Consider, for instance, one of the first and most popular scientific protocols for assessing fish welfare: the SWIM protocol for Atlantic salmon (*Salmo salar*) farmed in sea cages [31]. The SWIM 1.0 welfare protocol takes into account a number of welfare-related inputs and outputs: water temperature, salinity, stocking density, lighting, disturbance, daily mortality rate, appetite, sea lice infestation ratio, condition factor, emaciation state, vertebral deformation, maturation stage, smoltification state, fin condition, and skin condition [31,32]. Complementarily, SWIM 2.0 adds the need to attend to eye condition, cardiac condition, abdominal organs, gills, opercula, skeletal muscles, vaccine-related pathology, aberrant fishes, necropsy, and active euthanasia [33]. These types of indicators—including the ones in the most recent protocols available [34]—are validated tools for making inferences about the health of an individual. None of these, however, addresses Dawkins’s second question—are we giving animals something they want—let alone questions regarding what constitutes a good life for Atlantic salmon. Are these individuals able to express patterns of behavior they show strong motivation to perform? Are we providing them with resources they find valuable? Do they have the opportunity to make choices? Yet the SWIM protocol is a forward-thinking improvement on the total absence of protocols for the majority of farmed fish species [35]. The paucity of knowledge about fish welfare is a consequence of several key hurdles discussed below.

### 2.1. Underwater Lives 

Fishes are aquatic animals, and most species spend their entire lives underwater, making their behavior more challenging to observe than that of terrestrial vertebrates. For instance, although Atlantic salmon is one of the most studied farmed fish species, and we know that most Atlantic salmon migrate from freshwater to the ocean after smolting, we know very little about the crucial phase of their post-smolt lives that they spend in the oceans [36,37,38,39]. Despite this, these animals are among the most important farmed species. Although very far behind the fish species farmed in the largest numbers, such as Nile tilapia (*Oreochromis niloticus*) and some carp species, between 280 and 653 million of salmons were farmed in 2017 [3,4]. To account for the very different stages in a salmon’s life, farmed salmon are most often raised in land-based freshwater tanks and then transported to sea cages after smolting. However, this husbandry system does not accommodate their normal migratory behavior. Since variation exists both at the population and individual level [40] and some salmon do not migrate at all [41,42], we must consider the hypothesis that this activity is not necessarily a behavioral priority for salmon. However, to our knowledge, no one has investigated whether farmed salmon show a strong preference to migrate when given the choice. More importantly, no one has assessed the impact of being unable to migrate on salmon welfare. Salmon producers have not genetically selected salmon from non-migrating populations, so the possibility that the welfare of farmed salmon is negatively impacted by this behavioral restriction is viable. To further complicate husbandry decisions, we do not know what salmon need, much less what they want, in their ocean environment. Eel aquaculture is another example where migratory fishes are farmed without understanding what these animals need in order to experience good welfare. These aquaculture practices are depending on wild populations of glass eels (*Anguilla spp.*) that are caught while migrating upstream and kept in captivity. These are just two instances of how aquaculture has prematurely created husbandry systems—before securing the knowledge necessary to ensure good welfare.

### 2.2. Fish Hodgepodge: A Great Phylogenetic Variability

A second fundamental hurdle is that, from a phylogenetic viewpoint, fish species compose an immense group, characterized by great variability across the species under the umbrella concept of “fish” [43]. For instance, Actinopterygii, commonly known as ray-finned fishes, constitute half of all living vertebrate species [44]. Most ray-finned fishes are teleosts, and teleost species are dominant in marine and freshwater vertebrate faunas [45]. 

This vast phylogenetic variability has major implications for the study of welfare in fish species. Beyond sharing some very basic needs, such as food and dissolved oxygen, fishes belonging to different species have very specific needs that must be fulfilled in order to experience good welfare. As obvious as it seems, continued emphasis on this point is very important. Animal welfare scientists do not make imprecise generalizations regarding the welfare of various farmed terrestrial vertebrate species; they conduct specific research on swine, cattle raised for meat, cattle used for dairy, chickens raised for meat, and chickens farmed for eggs. For similar reasons, scientists must keep conducting species-specific research aimed at unveiling the needs and preferences of individual fish species [46]. After all, “what you call a fish may be as different as a mouse and a moose” [47].

In addition, it is crucial to address the specific needs related to each developmental stage these aquatic animals go through during their life cycles. Diadromous fishes, for instance, undergo very distinct life stages that typically take place in extremely diverse ecosystems. This is best exemplified by species with complex life cycles, such as the steelhead trout (*Oncorhynchus mykiss*), who in the wild typically migrate between freshwater and saltwater environments several times throughout their lives [48]. Thus, farming a species such as steelhead trout is particularly problematic. We must ask why these animals migrate between these very different environments. Is this a behavior inherent to good welfare, or do steelhead trout simply seek resources in different environments that could be provided in one? We do not know the answers to such questions. Accordingly, as with farmed terrestrial vertebrates, research on fish welfare must be specific not just to each species but to every life stage under farming conditions.

### 2.3. Fish Domestication?

Compared with domestication of terrestrial animal species, domestication of fish species is still in its early stages [49,50]. Indeed, although aquaculture is an ancient practice, domestication of most farmed fish species started only in the last century [51,52]. This has important consequences for the study of these animals’ welfare. Both our folk and scientific knowledge of most fish species are still poor, especially when compared with our knowledge of domestic terrestrial species. Moreover, knowledge acquired through observation and study of wild populations does not necessarily reveal the specific welfare needs of the same fish species as exploited in aquaculture, for farmed fishes have withstood strong artificial selection processes focused primarily on production-related traits [49]. In addition, key personality-related attributes that have proven essential for enhancing the welfare of terrestrial vertebrates under captivity, such as temperament, are not still a priority in farmed fish selective breeding [40].

### 2.4. Fish Cognition and the Sentience Controversy

Only recently have scientists started to delve deeper into the cognitive capabilities of fish species. The focus had been on fish cognition in general, which was similar to investigating mammalian cognition on the assumption that moose and mice see the world similarly. Species-specific efforts are not only essential to better understanding these animals’ worlds, needs, and preferences but to gathering key evidence to support a scientific answer to a fundamental question: Are fish sentient [53]? Despite the elaborated arguments against this notion [54,55], the great variability across fish species and our dearth of knowledge about most of them, current behavioral and neurophysiological evidence support the hypothesis that teleosts—and perhaps all fish species—process information at the conscious level and are capable of suffering from psychological stressors in addition to physiological stressors [56,57,58,59,60]. In light of this body of evidence, we should err on the side of caution and, as with other vertebrates, such as nonverbal mammals and birds, treat fishes as sentient animals whose specific welfare needs must be addressed.

## 3. Same Mistake Twice? Learning from Farmed Terrestrial Vertebrates

Even under the best available aquaculture practices, for the sake of financial profit, we risk repeating the mistakes that created serious welfare problems for farmed terrestrial vertebrates [54,61]. To shed light on this issue, let us reexamine research in laying hen welfare.

We now know that laying hens are strongly motivated to forage, a need they must fulfill to experience good welfare. Animal welfare scientists reached this conclusion by observing the behavioral patterns typical of this species and then asking themselves the right questions. They arrived at these determinations: (1) the provision of ad libitum feed does not eliminate hens’ natural need to forage, and (2) even in the presence of free food, hens choose to work in order to display foraging behavior, a concept termed “contra-freeloading” [62,63]. Through similar studies, inspired by behavioral observation (e.g., activity budgets) and cognitive findings [64], we have also learned that chickens are strongly motivated to perform many other patterns of behavior related to accessing larger areas and exploring [65], roosting [66,67], wing stretching [68], nesting [69,70,71,72], and dustbathing [73,74]. 

These studies have enabled us to better understand which environments provide these animals with resources and conditions they prefer and with opportunities to express patterns of behavior they are motivated to perform. Additionally, we have investigated the effects of each type of husbandry system (i.e., battery cages, enriched cages, cage-free facilities, and free-range facilities) on the animals’ physical health, taking into account the preferences that these animals have expressed. Importantly, these efforts have led to some significant regulatory changes, such as the Council Directive 1999/74/EC that effectively banned conventional battery cages in egg production systems in the European Union [75]. Only through this varied and extensive research can we now start making strong inferences on how to best provide these animals with conditions in which positive experience strongly outweighs negative—that is, with good lives. 

In light of this, scientists working on farmed fish welfare must avoid the serious mistakes made in connection with farmed terrestrial vertebrates. Indeed, this challenge is paramount. For instance, only after most of the egg industry moved hens into battery cages and most of the dairy industry moved cattle into zero-grazing systems did animal welfare scientists learn of the health and welfare risks associated with these behaviorally restrictive systems [76,77,78]. The pork industry increased productivity by moving gestating sows into crates without considering the risks to health and welfare [79]. Animal agriculture as a whole has made the mistake of using breeds genetically selected for increased productivity but not welfare [80,81,82,83,84]. 

The aquaculture industry has the advantage of a more complex understanding of animal welfare science to inform its growth. There is a great variability among aquaculture systems, some of which could potentially provide farmed fishes with good lives. However, in order to maximize profit, and potentially at the expense of positive welfare, current aquaculture practices are leaning towards intensification. With that trend, producers may be disposed to errors analogous to those of terrestrial animal agriculture, such as (1) preventing or disrupting expression of behavioral patterns key to enhancing these animals’ welfare [85], (2) not providing resources these animals show motivation for, let alone providing opportunities for choice and control in their environments [86,87], and (3) using breeds genetically selected to enhance productivity but prone to health and welfare problems [88].

## 4. Positive Fish Welfare: Unveiling What Fishes Need and Like

For more than a decade, there has been a gradual shift from classic paradigms focused mostly on improving farmed animal welfare by minimizing negative states—best exemplified by the influential Five Freedoms [89]—towards what is coined “positive animal welfare” [10,90,91]. This latter concept aims to maximize the positive aspects of welfare; thus, it is a more ambitious and challenging approach to investigating farmed animal welfare. Despite the noted challenge of making valid inferences regarding these animals’ various affective states and the relative novelty of the concept, the positive welfare framework has now been applied to farmed terrestrial vertebrates, such as chickens [7] and pigs [92]. However, we still lack comparatively robust, species-specific scientific knowledge about positive welfare in farmed fish. The scientific tools currently used to assess farmed fish welfare are still mostly focused on evaluating these animals’ physical health, which is inadequate for gathering the knowledge required to go beyond health and provide farmed fishes with positive welfare. Nevertheless, the investigation of welfare in fish species has seen clear advances. Several important publications covering these broader conceptions of welfare have emerged in recent years [93,94,95,96], including exciting empirical work on positive experiences in gilthead sea bream (*Sparus aurata*) [97] and novel behavioral indicators of welfare and positive emotion in zebrafish (*Danio rerio*) [98,99]. However, with rare exception [100], only very recently have animal welfare scientists started to pay attention to the challenge of investigating species-specific positive welfare in farmed fish species [9,101]. The publications examine the current evidence and provide areas for future research on this topic. The challenge remains monumental, and until we collect the necessary evidence, we must seriously consider the possibility that we cannot provide fish species with good lives under financially viable farming conditions. 

## 5. The Ultimate Frontier: Welfare as an Individual Attribute

Another crucial challenge is that welfare is an individual attribute, an idea more easily applied to animals in zoos [102,103]. While investigating welfare at the individual level is easy when dealing with, for instance, three grizzly bears, it is much harder—if not impossible—to consider individual welfare under, among other farming conditions, high stocking densities. Farmed animals are often forced to live in environments very different from those in which their species evolved. Aquaculture is no exception, and our goal is to understand which environmental conditions, stocking densities, and management practices can provide every individual with a good life.

To achieve positive welfare for every farmed animal, we should look to the welfare literature on animals in zoos, which focuses more heavily on behavior and physiological markers of stress as indicators of affect and, hence, welfare [104]. Despite the impossibility of investigating the welfare of each of tens of thousands of salmon in an open net pen, scientists have begun to devise sampling strategies that allow for welfare assessment that goes beyond health and focuses on the individual [105,106,107]. This research should be expanded and replicated in other farmed fish species, focusing primarily on which behavioral patterns are red flags for poor welfare (e.g., stereotypies) [108] and how these behaviors relate to physiological markers of stress [109,110]. We can continue to improve video behavioral monitoring tools [111,112] and combine behavioral data with glucocorticoid levels taken from water samples. As the knowledge on positive fish welfare increases, protocols such as Welfare Quality^®^—which include key behavioral assessment tools or can be used in tandem with qualitative behavior assessment, and have been developed to assess individual welfare in farmed terrestrial animals—should be adapted to measure a sample of farmed fish at the animal level [113]. The aim is to assess the range of welfare states on any one farm and determine how husbandry practices influence that range, and to do this for each species being farmed. 

## 6. Conclusions and Next Steps

The aquaculture industry is still in its infancy despite its rapid growth, and considering the hundreds of fish species now being farmed in multiple husbandry systems, the research on farmed fish welfare is still scant. We are at a pivotal point in the growth of this industry; we can look to the welfare literature on both farmed terrestrial animals and animals in zoos not only to avoid repeating mistakes but also to inform progress as the industry grows. Rather than work to enhance only productivity and health, fish welfare scientists can play a key role in working towards positive welfare for every farmed fish. 

## References

[1] Food and Agriculture Organization of the United Nations (2018). The State of World Fisheries and Aquaculture: Meeting the Sustainable Development Goals.

[2] Food and Agriculture Organization of the United Nations FAOSTAT Database. http://www.fao.org/faostat/en/.

[3] Food and Agriculture Organization of the United Nations Fisheries Global Information System, Global Aquaculture Production. http://www.fao.org/fishery/statistics/global-aquaculture-production/query/en.

[4] Fishcount Numbers of Farmed Fish Slaughtered Each Year. http://fishcount.org.uk/fish-count-estimates-2/numbers-of-farmed-fish-slaughtered-each-year.

[5] Broom D.M. (1986). Indicators of poor welfare. Brit. Vet. J..

[6] Dawkins M.S. (2008). The science of animal suffering. Ethology.

[7] Edgar J.L., Mullan S.M., Pritchard J.C., McFarlane U.J.C., Main D.C.J. (2013). Towards a “good life” for farm animals: Development of a resource tier framework to achieve positive welfare for laying hens. Animals.

[8] Mellor D.J. (2016). Updating animal welfare thinking: Moving beyond the “Five Freedoms” towards “a life worth living”. Animals.

[9] Fife-Cook I., Franks B. (2019). Positive Welfare for Fishes: Rationale and Areas for Future Study. Fishes.

[10] Lawrence A.B., Vigors B., Sandøe P. (2019). What is so positive about positive animal welfare? A critical review of the literature. Animals.

[11] Mattiello S., Battini M., De Rosa G., Napolitano F., Dwyer C. (2019). How Can We Assess Positive Welfare in Ruminants?. Animals.

[12] Mellor D.J. (2019). Welfare-aligned Sentience: Enhanced Capacities to Experience, Interact, Anticipate, Choose and Survive. Animals.

[13] Miller L.J., Vicino G.A., Sheftel J., Lauderdale L. (2020). Behavioral Diversity as a Potential Indicator of Positive Animal Welfare. Animals.

[14] Rault J.-L., Hintze S., Camerlink I., Yee J.R. (2020). Positive Welfare and the Like: Distinct Views and a Proposed Framework. Front Vet. Sci..

[15] Stokes J.E., Mullan S.M., Takahashi T., Monte F., Main D.C. (2020). Economic and Welfare Impacts of Providing Good Life Opportunities to Farm Animals. Animals.

[16] Vigors B., Lawrence A.B. (2019). What Are the Positives? Exploring Positive Welfare Indicators in a Qualitative Interview Study with Livestock Farmers. Animals.

[17] Ward S., Hosey G. (2019). The Need for a Convergence of Agricultural/Laboratory and Zoo-based Approaches to Animal Welfare. J. Appl. Anim. Welf. Sci..

[18] Webb L.E., Veenhoven R., Harfeld J.L., Jensen M.B. (2018). What is animal happiness?. Ann. N. Y. Acad. Sci..

[19] Makowska I.J., Weary D.M. (2020). A good life for laboratory rodents?. ILAR J..

[20] Seligman M.E.P., Csikszentmihalyi M. (2000). Positive psychology: An introduction. Am. Psychol..

[21] Lomas T., Ivtzan I. (2015). Second Wave Positive Psychology: Exploring the Positive—Negative Dialectics of Wellbeing. J. Happiness Stud..

[22] Meehan C.L., Mench J.A. (2007). The challenge of challenge: Can problem solving opportunities enhance animal welfare?. Appl. Anim. Behav. Sci..

[23] Franks B., Mench J.A. (2018). Cognition as a cause, consequence, and component of welfare. Advances in Agricultural Animal Welfare.

[24] Franks B., Higgins E.T., Olson J.M., Zanna M.P. (2012). Effectiveness in humans and other animals: A common basis for well-being and welfare. Advances in Experimental Social Psychology.

[25] Whitham J.C., Wielebnowski N. (2013). New directions for zoo animal welfare science. Appl. Anim. Behav. Sci..

[26] Špinka M. (2019). Animal agency, animal awareness and animal welfare. Anim. Welf..

[27] Spinka M., Wemelsfelder F., Appleby M.C., Mench J.A., Olsson I.A.S., Hughes B.O. (2011). Environmental challenge and animal agency. Anim. Welf..

[28] Purves D., Delon N. (2017). Meaning in the lives of humans and other animals. Philos. Stud..

[29] Franks B. (2019). What do animals want?. Anim. Welf..

[30] Broom D. (1988). The scientific assessment of animal welfare. Appl. Anim. Behav. Sci..

[31] Stien L.H., Bracke M.B.M., Folkedal O., Nilsson J., Oppedal F., Torgersen T., Kittilsen S., Midtlyng P.J., Vindas M.A., Øverli Ø. (2013). Salmon Welfare Index Model (SWIM 1.0): A semantic model for overall welfare assessment of caged Atlantic salmon: Review of the selected welfare indicators and model presentation. Rev. Aquac..

[32] Folkedal O., Pettersen J., Bracke M., Stien L., Nilsson J., Martins C., Breck O., Midtlyng P., Kristiansen T. (2016). On-farm evaluation of the Salmon Welfare Index Model (SWIM 1.0): Theoretical and practical considerations. Anim. Welf..

[33] Pettersen J.M., Bracke M.B., Midtlyng P.J., Folkedal O., Stien L.H., Steffenak H., Kristiansen T.S. (2013). Salmon welfare index model 2.0: An extended model for overall welfare assessment of caged Atlantic salmon, based on a review of selected welfare indicators and intended for fish health professionals. Rev. Aquac..

[34] Noble C., Gismervik K., Iversen M.H., Kolarevic J., Nilsson J., Stien L.H., Turnbull J.F. Welfare Indicators for Farmed Atlantic Salmon: Tools for Assessing Fish Welfare. https://nofima.no/wp-content/uploads/2018/11/FISHWELL-Welfare-indicators-for-farmed-Atlantic-salmon-November-2018.pdf.

[35] Saraiva J.L., Arechavala-Lopez P., Castanheira M.F., Volstorf J., Studer B.H. (2019). A Global Assessment of Welfare in Farmed Fishes: The FishEthoBase. Fishes.

[36] Lacroix G.L. (2013). Migratory strategies of Atlantic salmon (*Salmo salar*) postsmolts and implications for marine survival of endangered populations. Can. J. Fish Aquat. Sci..

[37] Byron C., Burke B.J. (2014). Salmon ocean migration models suggest a variety of population-specific strategies. Rev. Fish Biol. Fish.

[38] Ounsley J.P., Gallego A., Morris D.J., Armstrong J.D. (2019). Regional variation in directed swimming by *Atlantic salmon* smolts leaving Scottish waters for their oceanic feeding grounds—A modelling study. ICES J. Mar. Sci..

[39] Dadswell M.J., Spares A.D., Reader J.M., Stokesbury M.J.W. (2010). The North Atlantic subpolar gyre and the marine migration of *Atlantic salmon Salmo salar*: The ‘Merry-Go-Round’ hypothesis. J. Fish Biol..

[40] Gesto M., Villarroel M., Tort L. (2020). Fish individuality, physiology and welfare. Physiol. Behav..

[41] Guiry E.J., Needs-Howarth S., Friedland K.D., Hawkins A.L., Szpak P., Macdonald R., Courtemanche M., Holm E., Richards M.P. (2016). Lake Ontario salmon (*Salmo salar*) were not migratory: A long-standing historical debate solved through stable isotope analysis. Sci. Rep..

[42] Hutchings J.A., Ardren W.R., Barlaup B.T., Bergman E., Clarke K.D., Greenberg L.A., Lake C., Piironen J., Sirois P., Sundt-Hansen L.E. (2019). Life-history variability and conservation status of landlocked *Atlantic salmon*: An overview. Can. J. Fish Aquat. Sci..

[43] FishBase. https://www.fishbase.de/search.php.

[44] Friedman M. (2015). The early evolution of ray-finned fishes. Palaeontology.

[45] Near T.J., Eytan R.I., Dornburg A., Kuhn K.L., Moore J.A., Davis M.P., Wainwright P.C., Friedman M., Smith W.L. (2012). Resolution of ray-finned fish phylogeny and timing of diversification. Proc. Natl. Acad. Sci. USA.

[46] Kristiansen T.S., Fernö A., Pavlidis M.A., van de Vis H. (2020). The Welfare of Fish.

[47] Flik G. Fishing in an Underwater World?. Proceedings of the Fish Welfare Mini Symposium.

[48] Quinn T.P. (2018). The Behavior and Ecology of Pacific Salmon and Trout.

[49] Saraiva J.L., Castanheira M.F., Arechavala-Lopez P., Volstorf J., Studer B.H., Teletchea F. (2018). Domestication and welfare in farmed fish. Animal Domestication.

[50] Teletchea F., Pontarotti P. (2015). Domestication and genetics: What a comparison between land and aquatic species can bring. Evolutionary Biology: Biodiversification from Genotype to Phenotype.

[51] Fabrice T., Fontaine P. (2012). Levels of domestication in fish: Implications for the sustainable future of aquaculture. Fish Fish.

[52] Costa-Pierce B.A., Mann C., Costa-Pierce B.A. (2002). Archaeological Aquaculture. Ecological Aquaculture: Principles and Practices for the Blue Revolution to 2050.

[53] Fernö A., Folkedal O., Nilsson J., Kristiansen T.S., Kristiansen T.S., Fernö A., Pavlidis M.A., van de Vis H. (2020). Inside the Fish Brain: Cognition, Learning and Consciousness. The Welfare of Fish.

[54] Rose J.D., Arlinghaus R., Cooke S.J., Diggles B.K., Sawynok W., Stevens E.D., Wynne C.D.L. (2012). Can fish really feel pain?. Fish Fish.

[55] Key B. (2016). Why fish do not feel pain. Anim. Sentience.

[56] Braithwaite V. (2010). Do Fish Feel Pain?.

[57] Seth A.K. (2016). Why fish pain cannot and should not be ruled out. Anim. Sentience.

[58] Woodruff M.L. (2017). Consciousness in teleosts: There is something it feels like to be a fish. Anim. Sentience.

[59] Brown C. (2014). Fish intelligence, sentience and ethics. Anim. Cogn..

[60] Galhardo L., Oliveira R.F. (2009). Psychological stress and welfare in fish. Annu. Rev. BioMed. Sci..

[61] Santurtun E., Broom D., Phillips C. (2018). A review of factors affecting the welfare of Atlantic salmon (*Salmo salar*). Anim. Welf..

[62] Lindqvist C., Jensen P. (2008). Effects of age, sex and social isolation on contrafreeloading in red junglefowl (*Gallus gallus*) and White Leghorn fowl. Appl. Anim. Behav. Sci..

[63] Lindqvist C., Jensen P. (2009). Domestication and stress effects on contrafreeloading and spatial learning performance in red jungle fowl (*Gallus gallus*) and White Leghorn layers. Behav. Process..

[64] Marino L. (2017). Thinking chickens: A review of cognition, emotion, and behavior in the domestic chicken. Anim. Cogn..

[65] Dawkins M. (1977). Do hens suffer in battery cages? Environmental preferences and welfare. Anim. Behav..

[66] Campbell D.L.M., Makagon M.M., Swanson J.C., Siegford J.M. (2016). Perch use by laying hens in a commercial aviary. Poult. Sci..

[67] Olsson I.A.S., Keeling L.J. (2002). The push-door for measuring motivation in hens: Laying hens are motivated to perch at night. Anim. Welf..

[68] Nicol C.J. (1987). Behavioural responses of laying hens following a period of spatial restriction. Anim. Behav..

[69] Hughes B., Duncan I., Brown M.F. (1989). The performance of nest building by domestic hens: Is it more important than the construction of a nest?. Anim. Behav..

[70] Kruschwitz A., Zupan M., Buchwalder T., Huber-Eicher B. (2008). Nest preference of laying hens (*Gallus gallus* domesticus) and their motivation to exert themselves to gain nest access. Appl. Anim. Behav. Sci..

[71] Ringgenberg N., Fröhlich E.K., Harlander-Matauschek A., Würbel H., Roth B.A., Würbel H. (2014). Does nest size matter to laying hens?. Appl. Anim. Behav. Sci..

[72] Duncan I., Kite V. (1989). Nest site selection and nest-building behaviour in domestic fowl. Anim. Behav..

[73] De Jong I.C., Wolthuis-Fillerup M., Van Reenen C.G. (2007). Strength of preference for dustbathing and foraging substrates in laying hens. Appl. Anim. Behav. Sci..

[74] Guinebretière M., Beyer H., Arnould C., Michel V. (2014). The choice of litter material to promote pecking, scratching and dustbathing behaviours in laying hens housed in furnished cages. Appl. Anim. Behav. Sci..

[75] Directive E.U. (1999). Council Directive 99/74/EC of 19 July 1999 laying down minimum standards for the protection of laying hens. Off. J. Eur. Communities.

[76] Charlton G.L., Rutter S. (2017). The behaviour of housed dairy cattle with and without pasture access: A review. Appl. Anim. Behav. Sci..

[77] Crump A., Jenkins K., Bethell E.J., Ferris C.P., Arnott G. (2019). Pasture Access Affects Behavioral Indicators of Wellbeing in Dairy Cows. Animals.

[78] Shields S., Duncan I.J.H. (2009). An. HSUS Report: A Comparison of the Welfare of Hens in Battery Cages and Alternative Systems.

[79] Baxter E.M., Andersen I.L., Edwards S.A., Špinka M. (2018). Sow welfare in the farrowing crate and alternatives. Advances in Pig Welfare.

[80] Dawkins M., Layton R. (2012). Breeding for better welfare: Genetic goals for broiler chickens and their parents. Anim. Welf..

[81] Johnson P.A., Stephens C.S., Giles J.R. (2015). The domestic chicken: Causes and consequences of an egg a day. Poult. Sci..

[82] Oltenacu P.A., Broom D.M. (2010). The impact of genetic selection for increased milk yield on the welfare of dairy cows. Anim. Welf..

[83] Rutherford K.M.D., Baxter E., D’Eath R.B., Turner S., Arnott G., Roehe R., Ask B., Sandøe P., Moustsen V., Thorup F. (2013). The welfare implications of large litter size in the domestic pig I: Biological factors. Anim. Welf..

[84] Grandin T., Deesing M.J., Grandin T., Deesing M.J. (2013). Genetics and Animal Welfare. Genetics and the Behavior of Domestic Animals.

[85] Lindberg D.E. (2011). Atlantic Salmon (Salmo Salar) Migration Behavior and Preferences in Smolts, Spawners and Kelts; Introductory Research Essay.

[86] Maia C.M., Volpato G.L. (2018). Individuality matters for substrate-size preference in the Nile tilapia juveniles. J. Appl. Anim. Welf. Sci..

[87] Kagan R., Veasey J., Kleiman D.G., Thompson K.V., Baer C.K. (2012). Challenges of zoo animal welfare. Wild Mammals in Captivity: Principles and Techniques of Zoo Management.

[88] Fraser T.W.K., Fjelldal P.G., Hansen T., Mayer I. (2012). Welfare Considerations of Triploid Fish. Rev. Fish Sci..

[89] Farm Animal Welfare Council Five Freedoms. https://webarchive.nationalarchives.gov.uk/20121010012427/http://www.fawc.org.uk/freedoms.htm.

[90] Boissy A., Manteuffel G., Jensen M.B., Moe R.O., Spruijt B., Keeling L.J., Winckler C., Forkman B., Dimitrov I., Langbein J. (2007). Assessment of positive emotions in animals to improve their welfare. Physiol. Behav..

[91] Yeates J., Main D. (2008). Assessment of positive welfare: A review. Veter. J..

[92] Lawrence A.B., Newberry R.C., Špinka M., Špinka M. (2018). Positive welfare: What does it add to the debate over pig welfare?. Advances in Pig Welfare.

[93] Huntingford F.A., Adams C., Braithwaite V.A., Kadri S., Pottinger T., Sandøe P., Turnbull J. (2006). Current issues in fish welfare. J. Fish Biol..

[94] Ashley P.J. (2007). Fish welfare: Current issues in aquaculture. Appl. Anim. Behav. Sci..

[95] Huntingford F., Kadri S. (2014). Defining, assessing and promoting the welfare of farmed fish. Rev. Sci. Et Tech. De L’oie.

[96] Broom D. Fish Welfare and the public perception of farmed fish. Proceedings of the Second Nutreco Aquaculture Business Conference Stavanger Forum.

[97] Cerqueira M., Millot S., Castanheira M.F., Félix A.S., Silva T., Oliveira G.A., Oliveira C., Martins C.I.M., Oliveira R.F. (2017). Cognitive appraisal of environmental stimuli induces emotion-like states in fish. Sci. Rep..

[98] Deakin A.G., Buckley J., Alzu’Bi H.S., Cossins A.R., Spencer J.W., Al’Nuaimy W., Young I.S., Thomson J.S., Sneddon L.U. (2019). Automated monitoring of behaviour in zebrafish after invasive procedures. Sci. Rep..

[99] Franks B., Graham C., Von Keyserlingk M. (2018). Is Heightened-Shoaling a Good Candidate for Positive Emotional Behavior in Zebrafish?. Animals.

[100] Volpato G., Gonçalves-De-Freitas E., Fernandes-De-Castilho M. (2007). Insights into the concept of fish welfare. Dis. Aquat. Org..

[101] Martins C.I.M., Galhardo L., Noble C., Damsgård B., Spedicato M.T., Zupa W., Beauchaud M., Kulczykowska E., Massabuau J.-C., Carter T. (2011). Behavioural indicators of welfare in farmed fish. Fish Physiol. Biochem..

[102] Hosey G., Melfi V., Pankhurst S. (2013). Zoo Animals: Behaviour, Management, and Welfare.

[103] Kagan R., Carter S., Allard S. (2015). A Universal Animal Welfare Framework for Zoos. J. Appl. Anim. Welf. Sci..

[104] Hill S.P., Broom D.M. (2009). Measuring zoo animal welfare: Theory and practice. Zoo Biol..

[105] Roques J.A.C. (2013). Aspects of Fish Welfare in Aquaculture Practices. Ph.D. Thesis.

[106] Boerrigter J.G.J. (2015). Fish welfare: Adaptive Capacity of Farmed Fish. Ph.D. Thesis.

[107] Manuel R. (2015). Biology of Welfare in Fish Genes, Physiology and Behavior. Ph.D. Thesis.

[108] Mason G.J., Latham N.R. (2004). Can’t stop, won’t stop: Is stereotypy a reliable animal welfare indicator?. Anim. Welf..

[109] Roques J.A.C., Abbink W., Chereau G., Fourneyron A., Spanings T., Burggraaf D., Van De Bos R., Van De Vis H., Flik G. (2011). Physiological and behavioral responses to an electrical stimulus in Mozambique tilapia (*Oreochromis mossambicus*). Fish Physiol. Biochem..

[110] Galhardo L., Correia J., Oliveira R.F. (2008). The effect of substrate availability on behavioural and physiological indicators of welfare in the African cichlid (*Oreochromis mossambicus*). Anim. Welf..

[111] Kashiha M.A., Bahr C., Ott S., Moons C.P., A Niewold T., Tuyttens F., Berckmans D. (2014). Automatic monitoring of pig locomotion using image analysis. Livest. Sci..

[112] Rushen J., Chapinal N., De Passillé A. (2012). Automated monitoring of behavioural-based animal welfare indicators. Anim. Welf..

[113] Botreau R., Veissier I., Perny P. (2009). Others Overall assessment of animal welfare: Strategy adopted in Welfare Quality^®^. Anim. Welf..

